# Opportunities and challenges of delivering digital clinical trials: lessons learned from a randomised controlled trial of an online behavioural intervention for children and young people

**DOI:** 10.1186/s13063-020-04902-1

**Published:** 2020-12-09

**Authors:** Charlotte L. Hall, Charlotte Sanderson, Beverly J. Brown, Per Andrén, Sophie Bennett, Liam R. Chamberlain, E. Bethan Davies, Kareem Khan, Natalie Kouzoupi, David Mataix-Cols, Caitlin McKenzie, Tara Murphy, Mark Townsend, Chris Hollis, Elizabeth Murray

**Affiliations:** 1 NIHR MindTech Medtech Co-operative, School of Medicine, Division of Psychiatry and Applied Psychology, Institute of Mental Health, University of Nottingham Innovation Park, Triumph Road, Nottingham, UK; 2 University College London, Great Ormond Street Institute of Child Health, London, UK; 3 Centre for Psychiatry Research, Department of Clinical Neuroscience, Karolinska Institutet, & Stockholm Health Care Services, Region Stockholm, Stockholm, Sweden; 4 National Institute for Health Research, University of Southampton, Alpha House, Enterprise Road, Southampton, UK; 5 Research Department of Primary Care and Population Health, University College London, London, UK

**Keywords:** Randomised controlled trials, Internet, Online, Chronic tic disorder, Tourette syndrome, Recruitment, Retention, Research design

## Abstract

**Background:**

Despite being the gold standard of research to determine effectiveness, randomised controlled trials (RCTs) often struggle with participant recruitment, engagement and retention. These issues may be exacerbated when recruiting vulnerable populations, such as participants with mental health issues. We aimed to update understanding of the scope of these problems in trials of health technology and identify possible solutions through reflecting on experiences from an exemplar trial (Online Remote Behavioural Intervention for Tics; ORBIT).

**Method:**

We extracted anonymised data on recruitment, retention and requests for more funding and time from trials funded by the largest funder of health technology trials in the UK (the National Institute of Health Research Health Technology Assessment) between 2010 and 2020, and compared these with data from a recent, successful trial (ORBIT). ORBIT aimed to assess the clinical- and cost-effectiveness of blended online and human behavioural therapy for tics in young people. Many of the trial procedures, including recruitment, the intervention and data collection, were undertaken online.

**Results:**

Data were extracted on 51 trials conducted between 2010 and 2020. Sixty per cent of trials failed to reach their original recruitment target and only 44% achieved their follow-up in the specified time frame. In contrast, ORBIT recruited to target and achieved 90% follow-up. We posit that these achievements are related to (a) judicious use of digital technology for trial procedures and (b) adequate numbers of highly trained and motivated trial staff. We provide details of both these to help other research teams plan and cost for successful trials.

**Conclusion:**

An approach combining human and online methods may be advantageous in facilitating trial delivery, particularly in paediatric mental health services. Given the importance of successful clinical trials in advancing healthcare delivery and the waste of human and economic resources associated with unsuccessfully delivered trials, it is imperative that trials are appropriately costed and future research focusses on improving trial design and delivery.

**Trial registration:**

The ORBIT trial is registered with ISRTCN (ISRCTN70758207) Registered on March 20, 2018. and ClinicalTrials.gov (NCT03483493). Registered on March 30, 2018.

## Background

Randomised controlled trials (RCTs) are considered the ‘gold standard’ in research design for determining causality and assessing clinical- and cost-effectiveness of new health technologies or practices [1]. Evidence from high-quality (i.e. considered to be at low risk of bias) trials forms the basis for many clinical guidelines governing the delivery of care to patients (e.g., Scottish Intercollegiate Guidelines Network [2]) and is considered essential by organisations worldwide charged with determining which healthcare technologies should be funded such as the National Institute of Health and Care Excellence [3] in the UK and the Agency for Healthcare Research and Quality (AHRQ) in America [4]. However, RCTs require substantial resources and are often complex to design and deliver, as well as being demanding on both participants and research staff [5]. Thus, despite being considered the gold standard, these trials are prone to failure [6, 7], resulting in wasted resources, on both a time and economic level and raising ethical queries regarding the involvement of participants to no scientific advancement [8].

In recognition of the difficulties associated with conducting RCTs, research has examined factors associated with successful and unsuccessful trial delivery. One of the most pivotal studies conducted by Campbell et al. [9] was ‘STEPS’ (strategies for trial enrolment and participation study). Focussing on the issue of participant recruitment, the STEPS team found that less than one third of trials met their original recruitment target in time, and one third required a study extension. The study concluded that it was difficult to determine which factors were causally related to successful recruitment but noted that a good communication strategy, a dedicated trial manager and having interventions only available inside the trial were important factors. Trials which were deemed as ‘successful’ overall were conducted by well-regarded investigators and asked clinically important questions which were grounded in existing clinical practices. To date, the STEPS study still represents arguably the most comprehensive overview of challenges in trial recruitment.

Since then, there have been several attempts to define successful strategies for recruitment [5, 10] however, the majority have not been formally evaluated [11] or of limited success/lacking implementation [12]. Indeed, recent research confirms that recruitment remains a barrier to successful trial completion, with one paper showing that only just over half (56%) of trials met their recruitment target with or without a study extension [13].

Although receiving less attention in the literature, participant retention is also considered another significant threat to the success and validity of RCTs [14]. Whilst average retention in trials has been estimated at 89% [13], which may suggest that retention is less of a concern than recruitment, this figure is likely inflated by trials with only short-term follow-ups. Retention has also been shown to be particularly challenging in certain clinical groups or types of intervention, such as behavioural intervention trials involving participants with a mental health disorder [15]. Studies on smoking cessation for participants with depression or substance use disorders, for example, have reported follow-up rates as low as 27–33% [16, 17]. Though underlying factors behind poor retention are difficult to measure, participants report fatigue at completing lengthy assessments, or outcome measures that do not seem relevant to their condition or lived experience [18]. Poor retention has substantial implications to a trial, including, increasing study costs by requiring a larger sample size to achieve adequate power, and creating bias in results caused by attrition [19], particularly if there is a differential drop-out rate between group allocation which cannot fully be accounted for using statistical methods (such as multiple imputation) [15]. As such, researchers have also examined factors that influence good retention and found good communication which is adapted to suit the individual participant as well as regular reminders from trial staff to be beneficial [20].

Whilst clinical trials have been traditionally conducted in a clinical face-to-face setting, since the late 1990s, there has been an increasing trend towards online or digital trials [21], in which either the intervention and/or the outcome measures are collected remotely. Although the number of trials investigating an online intervention has increased over time, the number of online interventions is proportionately low to the number of trials being conducted, with mental health studies being one of the most prevalent fields [22]. Online delivery of trials is intuitively attractive, offering the ability for participants to self-refer, standardise the delivery of interventions, and allow participants a time-and-location convenient option to complete outcome measures [14, 23, 24].

There is mixed evidence regarding issues of recruitment and engagement with online trials. Whereas some trials have reported particularly good recruitment and engagement (e.g. [25]), other evidence indicates that online trials may be particularly susceptible to poor recruitment, limited engagement with the intervention [26] and higher drop-out rates [27, 28]. A recent systematic review indicated that trials of web-based interventions often fail to appropriately account for the level of intervention use (i.e. sessions completed) [22], indicating that the general acceptability of online interventions is not yet fully known. Some known possible barriers to the delivery of online trials include poor technology skills, interfaces that are not user-friendly, concerns around data security and a lack of support from healthcare professionals [26, 29].

An ongoing trial investigating the online delivery of behavioural therapy for tics (ORBIT trial, [30, 31]) has been particularly effective in recruiting and retaining participants. Consideration of methodological and design factors that may have contributed to this success may offer a helpful learning opportunity for future trials. The trial is a parallel-group, single-blind RCT which included an internal pilot phase and was funded by the NIHR Health Technology Assessment (HTA) (Ref 16/19/02) and ethically approved by North West Greater Manchester Research Ethics Committee (Ref 18/NW/0079). The trial recruited children and young people (aged 9–17 years) with a tic disorder. Participants were randomised to receive either an online, therapist supported behavioural intervention for tics or psychoeducation around tics. The trial used a ‘blended’ approach to delivery, combining a mix of online (web-based) procedures and procedures that were delivered, or supported by, trial therapists and staff (either face-to-face or via videoconferencing). The trial achieved the aims of the internal pilot which were set within the first 9 months of recruitment, with clear stop/go criteria to determine progression to a full definitive trial. The ORBIT trial continued to finish recruitment to time-and-target, maintaining follow-up rates at the primary end point that exceeded the 80% target, indicating potential benefits of interventions with online delivery.

This article aims to highlight some of the key risks in trial delivery and outline some of the trial management and conduct process that we believe were pivotal to the ORBIT trial success in achieving recruitment and retention targets. These learned experiences may help research teams inform their design of future trials, with specific focus on how online delivery may overcome some common pitfalls in trial delivery.

## Methods

### Design

Case study, comparing data from one specific trial (ORBIT trial, [30]) with RCTs funded by the same funder (the largest funder of health technology trials in England) over a 10-year period.

### Setting

The National Institute of Health Research (NIHR) is Europe’s largest funder of health and care research. In 2017–2018 its total budget was over £1billion; £252 million was allocated to individual research projects, of which £78.1 million was disbursed through the Health Technology Assessment Programme (HTA), responsible for funding evaluations of new health technologies, including pharmacological and non-pharmacological interventions [32]. As the funder of our case study, and the largest funder of health technology assessment studies in England, we deemed this the most suitable source to identify comparator trials.

### Search strategy for comparator trials

We limited our search to the most recent decade (2010–2020), to allow for learning from the influential STEPS study [9]. The following inclusion criteria were developed to ensure we identified all appropriate comparator studies: (1) recruited participants with a mental health/behavioural condition, as classified by ICD-10 [33]; (2) used a RCT design (feasibility and pilot RCTs were included); (3) reported a psychological or behavioural intervention (diagnostic interventions or changes to the care system were also included); and (4) the trial was classified by the HTA as completed. Only completed trials were included to reduce data skew from trials still in the recruitment phase but yet to achieve their specified targets. Trials classified as CTIMP (Clinical Trial of an Investigation Medicinal Product) were excluded as it is possible that recruitment and retention to a drug vs behavioural/psychological intervention trial may involve differential barriers and strategies.

A member of the HTA staff identified all studies funded between January 2010 to January 2020 which were coded as ‘mental health’ and/or ‘neurological’. Two members (CLH, CM) of the ORBIT study team independently reviewed the study titles and summaries against the inclusion and exclusion criteria. Any disagreements were resolved via discussion until consensus was reached. Data from the final list of included studies was provided anonymously by the HTA, with no reference to potential identifying information such as start/end dates or condition.

### Data extraction

Data on recruitment, retention to follow-up, and requests for variations to contract (either more time to complete the study, more financial resource or both) were extracted from anonymised progress and performance reports submitted to the funder by the Principal Investigators of included trials. Timely submission of such reports is a requirement of the funder, and release of funds is dependent on receipt of these reports. It was not possible to extract information about whether trials were conducted online or not as this is not an HTA reporting requirement, and as reports were anonymised, we could not cross-check them with published protocols. The HTA also do not record engagement with the intervention as a reportable criteria. However, in light of the recent systematic review indicating the need for greater understanding and reporting of engagement with online interventions [22], we have specifically outlined the ORBIT processes that we consider may have promoted engagement and treatment completion, although it is not possible to contrast this with other HTA trials.

### Data analysis

Each trial was coded as to whether it met recruitment and follow-up rates within the specified time frame or whether a variation to contract (i.e. study extension) was requested and granted. The primary reason for requesting a variation to contract was also coded. Descriptive statistics (number and percentage of trials) are presented for each criterion.

We then draw comparisons to the case study, ORBIT, highlighting key trial design and management processes that may have been influential in achieving the key targets. These key processes and reflections were generated via a focus group consisting of 14 key members of the ORBIT team management group. This management group included representatives from trial researchers, trial therapists, the clinical trials unit, the trial manager, principal investigators, international collaborators in Sweden and the chief investigator. The discussion was led by the trial manager who had generated initial topics for discussions based on the influential STEPS [9] paper. Reflections were recorded via typed minutes and reviewed and approved by the team for accuracy.

## Results

One hundred and seventy six studies funded by the HTA between 2010 and 2020 were classified as ‘mental health’ or ‘neurological’. Fifty one of these met the inclusion criteria.

### Recruitment

Of the 51 studies identified, one had no specified recruitment target. Attainment of recruitment targets for the remaining 50 studies are presented in Table 1 and shows that only 20 (40%) studies met their original recruitment target in time, one of which finished recruitment 3 months ahead of schedule. Twenty-three (46%) studies were given a revised target which was achieved in 61% of cases. Reasons for not meeting the target were not generally specified, although in one study, it was noted there was a 6-month delay in initially starting recruitment; however, after a 10-month extension, the study still did not meet the target.

**Table 1:** Number of studies meeting recruitment targets (*n* = 50)

	Met initial target (***n*** = 50)	Met revised target (***n*** = 23)
**Yes**	20 (40%)	14 (61%)
**No**	30 (60%)	9 (39%)

One study had no specified recruitment target and thus not included in the table

Seven studies that did not meet the initial target were not given a revised target for various reasons including: not feasible to continue (*n* = 2), reason not clearly specified (*n* = 2), safety issues (*n* = 1), better attrition rate than anticipated (i.e. still sufficient power) (*n* = 1), contributing to international study which met overall target (*n* = 1), conclusions could be drawn from existing sample (*n* = 1).

### Retention to follow-up

For the purpose of this paper, ‘follow-up’ refers to achieving the pre-specified target for participant retention to the primary outcome at the primary end point. From the 51 HTA studies, follow-up data was only available from 34 studies (67%). This missing data was due to historical limitations with the HTA recording systems. Table 2 presents the number of trials that met their pre-specified retention follow-up targets and shows that only 15 (44%) met their initial target. Revised time periods for data collection were given to three studies, resulting in one additional study meeting its target (47% of the 34 studies). Reasons for not meeting follow-up targets were not specified.

**Table 2:** Number of studies meeting follow-up targets (*n* = 34)

	Met initial target (***n*** = 34)	Met revised target (***n*** = 3)
**Yes**	15 (44%)	1 (33%)
**No**	19 (56%)	2 (67%)

In one case, the follow-up was underestimated from the start but the study was allowed to continue without a revised target. This has been categorised as ‘not meeting initial target’

### Requests for more time, more funds or both (variations to contract)

Table 3 displays the number of formal requests for variations to funding contracts. Variations to contracts typically involved requests for additional funds, time or both in order to complete the trial. The most common reason (found in 54% of trials) for requesting a variation to contract was due to issues with participant recruitment. The length of extensions requested due to issues with recruitment ranged from 2 to 22 months.

**Table 3:** Number of studies (*n* = 51) requesting at least one variation of contract (additional funds, time or both) by reason/issue

Reason for request	Type of request					Number of approved requests for funds
	Funds	Duration	Funds and duration	Other	Requests	
**Recruitment issues**	1	9	10	0	*N* = 20 Requesting funds =11	11/11
**Retention/follow-up issues**	1	2	1	0	*N* = 4 Requesting funds = 2	2/2
**Staff issues/volume of work**	1	0	4	0	*N* = 5* Requesting funds = 5	3/5
**Other/not clearly specified**	2	1	2	3	*N* = 8 Requesting funds = 4	4/4

Seven studies requested more than 1 variation to contract

*Two of the 5 staff issues were linked to issues with recruitment

Notably, 5 out of the 51 studies requested a variation to contract due to issues relating to staff (see Table 3). This was responsible for 13.5% of variation to contract requests. Out of these 5, a further breakdown of the reasons showed that 2 cited the volume of work (1 specifically linked to recruitment), 1 maternity leave, 1 maternity leave and combined issues with recruitment and 1 had no further details. Issues with staffing is not a reportable criterion for HTA studies unless the trial team are requesting a variation to contract. Thus, it is not possible to understand the full extent of trials that are reporting difficulties due to staff/workload which is impacting on trial delivery.

### ORBIT case study

**Fig. 1:**
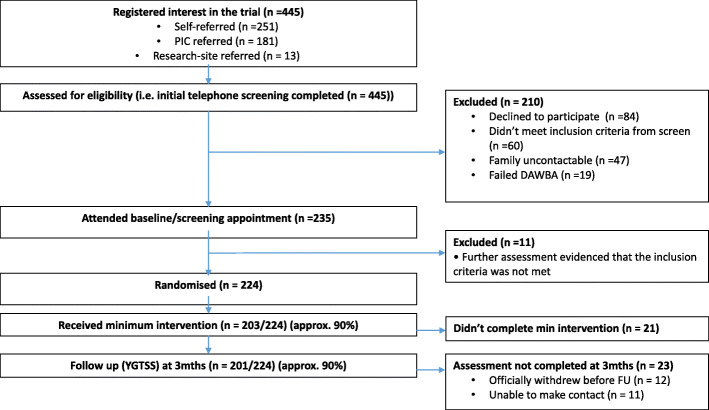
ORBIT study flow. DAWBA, Development And Well-Being Assessment given at screening to determine eligibility; PIC, patient identification centre

A summary of the ORBIT study flow is presented in Fig. 1. Recruitment and retention targets and attainment are shown in Table 4. To determine progression to a full trial, the first 9 months of the trial included an internal pilot with key targets. Table 4 shows the attainment of targets for both the internal pilot and full trial. The required study sample was 220 participants which was powered to detect a clinically important average difference of 0.5 standard deviation between intervention and comparator with 90% power at *p* < 0.05 (two-sided), after allowing for 20% drop-out [30].

**Table 4:** Key targets and attainment in ORBIT

	Target	Actual
**Recruitment**		
**Internal pilot**	66 participants by 9th month	67 participants by 6th month
**Full trial**	220 by 18th month	224 by 18th month
**Engagement with the intervention**		
**Internal pilot**	60% of participants classified as treatment completers by 9th month	96% participants classified as treatment completers by 6th month
**Full trial**	Not specified	90.6% completed
**Retention to primary end point**		
**Internal pilot**	80% retention by 9th month	88% retention by 6th month
**Full trial**	80% retention	90% retention

_Treatment completers were specified a prior as completion of the first 4/10 therapy chapters_

**Fig. 2:**
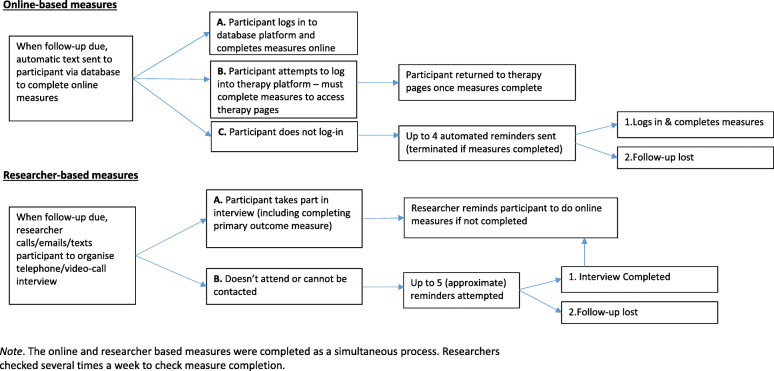
ORBIT process for obtaining follow-up measures. The online and researcher based measures were completed as a simultaneous process. Researchers checked several times a week to check meausure completion

Recruitment to ORBIT encompassed three modes of recruitment: online self-referral, clinical research sites and participant identification centres. ORBIT followed-up participants at 3 months post-randomisation (primary end point, just after completion of the intervention), and then again at 6, 12 and 18 months post-randomisation. Follow-ups comprised online self-report measures collected via an online database developed by the Karolinska Institutet eHealth Core Facility with automated and researcher controlled functions and a video-conference interview with the study researcher. A brief overview is shown in Fig. 2.

The ORBIT trial met the internal pilot recruitment targets ahead of schedule and overall recruitment finishing to time and target. The trial exceeded both its internal pilot target and final follow-up target at the primary end point (3 months). At the time of publication, longer-term follow-ups were still ongoing.

### Potential reasons for recruiting to target

We considered that the following factors were pivotal to successful recruitment:

1. *National recruitment:* Provision of mental health services is not evenly distributed; thus, there may be greater uptake of an intervention in under-provided geographical areas. Furthermore, some National Health Service (NHS) Trusts are well established in supporting research; in ORBIT, referrals from Patient Identification Centres (PICs) ranged from 0 to 27. The PICs were identified either via existing connections held by the study team or via the UK Clinical Research Network (CRN) database which lists active NIHR funded research for interested sites to contact the name investigator. Given the sites were not involved in the delivery of the intervention and thus were viewed as low-involvement sites by the study team, no feasibility checks were conducted. By having a large recruitment area, particularly for disorders with a lower prevalence rate, the trial was less affected by the underperformance from an individual region or trial site.
2. *Self-referrals*: The majority of ORBIT participants self-referred online, via a national charity ‘Tourettes Action’ and the study webpage hosted by the Institute of Mental Health, University of Nottingham. Allowing self-referrals enabled participants who were not currently under the care of a mental health service to be included. Self-referrals were particularly useful in the early stages of recruitment when NHS sites were slow in embedding the identification process in their workload and there were frequent hold-ups in gaining local regulatory approvals across the PICs.
3. *Unmet need for trial intervention:* ORBIT or similar online interventions were not freely available outside the trial for the UK. Furthermore, access to standard face-to-face behavioural therapy for tics is scarce with only 1 in 5 people having access to evidence-based treatment [34]. Conversely, there may be pragmatic barriers to recruiting participants in a specialist centre for the disorder where there are already established treatments available. As evidenced in ORBIT, the two research centres were specialist Tourette syndrome centres and only referred 13 patients (2.9% of referrals) into the trial.
4. *Patient and public involvement:* ORBIT recruitment documents were co-developed with an involvement group of children and young people with Tourette syndrome or chronic tic disorders and their parent/carers. The group informed on use of language, length of documents and layout, including incorporating different versions of information sheets for younger and older children. Additionally, the research team produced monthly short ‘spotlight on the researcher’ video-blogs and animated recruitment videos, which were hosted on Tourettes Action’s webpage. These videos engaged families with the research and resulted in spikes in self-referrals after each post.
5. *Regular monitoring and communication:* The trial manager tracked recruitment from each site and produced monthly newsletters to PICs identifying ‘star recruiters’, promoting both the concept of collaborative efforts to a shared goal as well as inter-site competition. Each PIC had regular fortnightly communication with the Trial Manager to promote engagement, build rapport and problem solve specific issues where necessary.
6. *Reimbursement and early exclusion*: As participants were required to travel across England for a baseline assessment at one of the two research centres, their travel costs were reimbursed by the study team. The initial telephone screen prior to this appointment enabled researchers to exclude prospective participants who clearly did not meet inclusion criteria to save patient and research time.

### Potential reasons for retaining to target

We believe the following factors were critical to outcome measure completion:

1. *Online outcome measures:* Automated reminders sent via the database directly to the participant. Additionally, these online outcome measures allowed participants to directly enter their data into the database and streamlined researcher time.
2. *Tokens of appreciation:* Participants were given £20 for completion of the outcome measures at each time point. Khadjesari et al. [35] note that tokens of appreciation of sufficient monetary value may also promote completion of online measures; however, ethics committees may be mindful of potential financial coercion. This amount may arguably not be seen as large enough to warrant coercion but sufficient to keep participant interest.
3. *Building a rapport:* ORBIT researchers often dedicated additional time in their online interviews to listen to the family struggles and successes, although they were careful not to offer advice outside the constraint of the trial. Where required, the researcher would send a standard approved template letter to the child’s general practitioner (GP) or school to signpost the potential need for assessment or further support. Where possible, the same researcher conducted baseline and all follow-up assessments, which also promoted consistency on measures that were subjectively rated by the researcher. The researcher also recorded important individual factors for each family (i.e. name of pet or preferred hobby). It should be noted, this personalised information was stored against their anonymous participant ID, separate from their name, address and date of birth, in a secure, password-protected file, accessible only by the research team. Where researchers failed to make contact the ORBIT therapist was sometimes asked to contact with the families if they had established a particularly strong rapport during treatment.
4. *Flexibility:* ORBIT researchers conducted follow-up interviews outside of normal working hours (such as evenings and weekends) to provide flexibility, ensuring that participation in the trial did not impact on the families school/work commitments. Although time of appointment was not recorded, our researchers estimate from reviewing available information in their diaries, that approximately 90% of appointments took place outside a typical school day, during the evening or weekend. Additionally, where families were unable, felt uncomfortable or experienced significant challenges using video-conferencing, telephone meetings were offered as an alternative to improve participant experience.
5. *Regular monitoring and communication:* The trial manager and researchers monitored retention rates on a monthly basis. The team discussed retention strategies and problem-solving.

### Characteristics of the ORBIT interventions

In the ORBIT trial, both arms received a therapist guided, online intervention for tics. One received a behavioural intervention based on exposure and response prevention principles trialled in Sweden (‘BIP TIC’) [36], and the other received psychoeducation developed by the ORBIT study team based on the intervention developed by Piacentini et al. [37]. Engagement with the ORBIT intervention exceeded expectations. The internal pilot specified that 60% of participants had to have completed treatment, the actual number completing was 96%. Overall, treatment completion for the trial was 90.6%. We consider the following factors to have been instrumental in influencing this positive uptake:

1. *Poor current provision:* As discussed in recruitment, access to evidence-based behavioural therapies in current care for this population was poor.
2. *Active control:* Both groups received an active treatment that was likely to be more than they would be offered in standard care in most centres. Indeed, even in a specialist tic treatment centre, some young people may be offered psychoeducation (ORBIT active control) rather than behavioural therapy if that was felt to meet the needs of the young person best. At this current time, blinding codes have not been broken thus we are unable to comment on differences between arms; however, with 90% completion rate, it is unlikely that there would be a significant difference in engagement.
3. *Remotely delivered:* The content of the intervention was delivered remotely enabling families to log-in and complete the therapy at a time and place most convenient to them. Although the therapist would only respond or comment during standard working hours, usually, this did not stop families in continuing to progress.
4. *Parent/carer support:* Carers were actively engaged with the intervention to enable them to act as a ‘supporter’ for their child’s treatment. The supporters were provided with their own chapters which gave information as to how to support the child and the supporter played a key role in setting goals and rewards as part of the intervention. The therapists noted that typically the level of supporter involvement was an influential factor in predicting the child’s engagement, particularly for younger children.
5. *Therapist support:* In ORBIT the main therapeutic content was delivered via the online platform. As such, the therapist’s role was to promote adherence and motivation to the treatments, alongside setting goals and reviewing goal attainment. Although the therapist communications were primarily through the online platform (telephone contact was arranged, if requested), participants were introduced to their therapist during the face-to-face baseline appointment where possible. This was done to promote treatment credibility and encourage a rapport with the therapist. Similar to the researchers, where possible, the family were in contact with one therapist who remembered individual information such as interests of the child, to build rapport. Instances where the therapist went on leave, this information was shared with the covering therapist so that they could continue the established relationship.
6. *Research-supported infrastructure*: Conducting research in under-funded child mental health services where there is inadequate infrastructure to support additional research tasks is an additional barrier. In ORBIT, the therapist was provided, trained and closely supervised by the research team, reducing impact on the referring clinicians’ workload.

### ORBIT research staff

The HTA data indicated that staffing issues were a key factor in requiring variations to contracts. ORBIT had two dedicated full-time researchers, one based at each of the two research centres.

Primarily, the researchers’ role was to assess eligibility, enrol participants into the trial, conduct baseline and follow-up assessments and report any adverse events to the trial manager. We reflect on the following factors that were important for ORBIT researchers:

1. *Peer support:* Although the two main researchers were located at different sites, they shadowed each other and provided peer-support which was aided by the trial manager. Monthly conference calls between the sites provided set time for shared learning experiences. The trial manager conducted weekly checks on each sites’ performance and offered support, advice or encouragement where needed. Additionally, ORBIT benefited from collaborating with the Swedish team at Karolinska Institutet that developed a first version of the active intervention tested in the ORBIT trial. This team also co-developed the database for outcome measures. Having easy access to staff at the Karolinska Institute for technical support and to aid troubleshooting was extremely important for the trial delivery.
2. *Flexibility:* As discussed previously, the two researchers provided appointments outside normal hours, including evening and weekends. This involved substantial ‘good will’ from the researchers and without this flexibility it is unlikely that the retention to follow-up would have been so high.
3. *Continuity:* Where possible the same researcher undertook both baseline and follow-up assessments.
4. *Early identification of training:* Undertaking trials is a complex procedure, with various standard operating procedures and guidelines which must be adhered too. Completing this training can take a significant amount of time which may impact on when a researcher is able to start actively enrolling participants into the trial. Appropriate time should be built in to grant proposals to allow for adequate researcher identification and training.
5. *Additional funding:* The time taken to undertake each outcome measure is not a simple sum of the time taken to administer the measure. Additional tasks such as following up on adverse events, sending letters to GPs or schools, rebooking if families did not attend appointments, data entry and responding to queries all added a significant burden to researcher time that was not costed for. ORBIT was only able to stay on track due to additional NIHR infrastructure support provided by NIHR MindTech MedTech Co-operative in the form of both staff time and funding. An additional part-time researcher was bought in during the first 4 months of recruitment to facilitate screening across both sites, the costs for this were not provided by the HTA trial grant but were provided by the NIHR MindTech MedTech Co-operative. Additionally, ad-hoc support was provided by a PhD student.

A summary of the challenges and opportunities learned via ORBIT is presented in Table 5.

**Table 5:** Summary of challenges and opportunities from the ORBIT trial

Challenge	Solutions and opportunities
*Recruitment*	National (or geographically large scale) recruitment
	Self-referrals (reduce reliance on clinical referrals)
	Intervention meets an unmet need
	Patient and public involvement on design and patient facing documents
	Regular monitoring and communication with recruiting sites
	Reimbursement for participant travel and early exclusion prior to attending a face-to-face appointment
*Retention*	Online outcome measures
	Participant tokens of appreciation
	Building participant rapport and patient and public involvement in study design
	Flexibility in completing follow-up interviews outside normal office hours
	Regular monitoring and communication with trial staff
*Engaging with the intervention*	Poor current provision of care in the area of interest
	Active control intervention
	Intervention remotely delivered
	Parent/carer actively involved
	Therapist support
	Research-supported infrastructure (research teams provide require staff/training)
*Research staff*	Peer support
	Flexibility in working pattern
	Continuity
	Early identification of training
	Additional funding

## Discussion

With the aim of updating and building upon the pivotal STEPS [9] study and providing researchers and funders with a resource to inform future trial design and delivery, we presented data on current recruitment and retention rates in trials funded by the HTA, a large UK funder. These HTA data demonstrated that less than half of trials of psychological/behavioural interventions between 2010 and 2020 delivered on key targets recruitment and retention. Comparatively, ORBIT (a trial of a remotely supported behavioural intervention) recruited to time and target and achieved 90% follow-up. We consider that the careful use of technology blended with well trained and motivated staff were key in achieving this and also in facilitating participant engagement with the intervention.

The HTA data demonstrated that only 40% of trials reviewed met their initial recruitment target, and issues with recruitment were the single biggest factor (54%) for not completing trials to time-and-target. Our findings are comparable to the STEPS [9] study and a more recent study of published HTA trials [13] who estimated that approximately 30–50% of trials met their recruitment target. Although it is not possible to make a direct comparison on trial design/population with the STEPS paper, it is interesting that over 10 years later, recruitment still remains a significant barrier in successful trial completion. Similarly, in line with previous studies, we also found evidence of poor retention rates in trials of psychological interventions [15]. Only 47% of the studies achieved their specified follow-up rate even with extensions, indicating that the majority of trials were potentially under-powered. However, issues with follow-up were less frequently cited for reasons for requesting funding extensions. It should be noted that follow-up data was not available for 17 of the 51 studies due to historical differences in HTA systems for record keeping, as such it is possible that our findings may not accurately represent the full picture.

Although limitations in HTA standard reporting precluded comparisons of intervention engagement between trials, we considered it was important to reflect on the intervention engagement in ORBIT as this was likely a factor in subsequent retention. Non-adherence to the intervention is a common problem in RCTs, with intervention non-adherence ranging from 2 to 78% in drug and psychological/behavioural intervention trials, with a median of 38% non-adherence [38]. Furthermore, a recent systematic review revealed that treatment adherence is particularly overlooked in internet-based trials. For example, Koneska et al. [22] found that although 90% of trials of an internet-delivered intervention collected usage data, only 39% investigated the level of intervention use and only 21% used statistical methods to account for this differential usage in the analysis. Without presenting information on intervention completion, it is difficult to know if the intervention itself is not effective and estimate intervention specific effects on retention [22]. Furthermore, although the trial methodological processes were undoubtedly important in promoting recruitment and engagement, the success of the trial was also based on offering an attractive intervention which was, otherwise, unavailable thereby addressing an unmet need. The acceptability of the ORBIT intervention is currently being explored via a process evaluation, including a qualitative component of participants’ opinion and experiences [39].

Due to restrictions on the granularity of detail of the HTA, it is not possible to know the specific characteristics or reasons why many studies did not meet their initial targets. For example, it would have been interesting to have been able to examine differences across participant conditions/characteristics, or issues that may have arisen with study set-up, or how many studies offered monetary incentives to participants. It was also not possible to distinguish between performances of online or non-online delivered trials from the HTA data set, although it is likely that different types of trial delivery have their own set of challenges. Indeed, some previous studies indicate that online trials are susceptible to specific challenges such as potential breaches to confidentiality through online communication [40] as well as lack of personalisation and difficulties with rapport building with participants [41]. Furthermore, it is likely that conducting trials with any online element may be particularly problematic in elderly or very deprived populations, with poor internet access and/or lack of privacy, and thus, we are not advocating online delivery as a blanket approach. For children and young people, delivering online interventions in school/ colleges setting may mitigate some of these access limitations associated with the home. However, utilising the ORBIT trial as an example, we illustrate how online delivery of interventions and outcome measures may help increase the geographical reach for recruitment by avoiding costly and time-consuming visits to clinic for both participants and researchers/therapists. Online interventions may also aid intervention engagement for some, by allowing flexibility to complete treatments from home at evenings or weekends. Greater standardisation of procedures using online delivery also has the potential to reduce cross-contamination, which is a particular risk in standard face-to-face trials where therapists deliver multiple interventions. Furthermore, online delivery of interventions may be more cost-effective, reducing the need for highly-skilled therapists. Finally, completing outcome measures online directly into trial databases with automated reminders for completion is likely to reduce burden for researchers, data-entry time and errors and may promote greater completion of measures.

Although on the surface it may seem tempting to rely solely on online methods for delivery of both interventions and outcome measures, we also highlight the key role that research staff play in promoting good recruitment and retention rates. It should also be noted that the ORBIT intervention integrated remote, therapist support. It is notable that issues with staffing were a recurrent reason for requests for variations to contracts in the HTA data. Though these reasons are not specified in more detail, continuity in research staff in ORBIT was identified as an important factor in promoting retention, particularly in building an ongoing rapport with participants. Previous studies have also indicated the importance of good staff communication [20] and that high staff turnover is associated with lower participant adherence [42], but also in a cyclical manner that difficulties in recruitment and retention of participants can reduce staff moral [19], which may in turn lead to staff turn-over.

ORBIT researchers also worked highly flexible work-patterns which included out-of-hours appointments to gather face-to-face outcome measures, to bolster the flexibility of online delivery for families taking part. As such, they encompassed the benefits of flexibility associated with online delivery but equally as important were able to build a rapport with participants. It should be noted that the flexible work-patterns may increase strain on research staff and although online delivery may go some way in reducing some of the demands associated with outcome measure completion (i.e. reduce data entry and automated reminders), greater investment is needed to understand how we can best recruit and retain/support staff as well as participants. We also note that research staff time should be appropriately costed, with the time taken to complete outcome measures being more than the sum total of minutes to deliver each item. Additional time is needed for rapport building (i.e. conversation with families), non-attendance and repeated attempts to make contact. Furthermore, as evidenced by the HTA data, long-term staff leave (i.e. sickness or maternity) can represent a significant threat to trials and there is benefit in having ‘bank researchers’ who are trained in the trial procedures and with the necessary permissions to provide immediate cover when required. The demand for staff time can vary across the lifespan of the trial—with particular pressures at various phases (e.g. initial recruitment/ enrolment and at follow-ups before the first participants leave trial). Although research staff costs typically represent a significant proportion of research funds, it is important that this is adequately costed to facilitate successful delivery. On reflection, we consider the ORBIT trial was under costed and the provision of additional staff was only possible with support from co-located NIHR infrastructure. This additional support is unlikely to be available to most funded trials. Although staff time is arguably one of the most expensive aspects of grant bid, funding bodies need to consider this cost balanced against the cost of partially-powered studies that have been unable to recruit/retain to target or that have required costed extensions to contracts.

Our experience of conducting research in child and adolescent mental health services in the UK indicates that these services often do not have appropriate infrastructure to support research delivery. We consider one of the key strengths of ORBIT was that therapists were identified, trained, closely supervised and employed by the research team, reducing the strain on already over-burdened healthcare systems. This was facilitated by the online delivery and therapist-supported self-help design of the intervention which allowed few therapists to support a large number of participants across a large geographical region. For example, ORBIT therapists supported up to 30 patients at one time, whereas for clinicians providing traditional face-to-face individual delivery of tic treatment this would likely be a much smaller caseload. Although online interventions can also be delivered without any therapist support, substantial research evidence indicates that therapist supported interventions promote better adherence than self-directed [43], and we consider this blended approach of human and online delivery to be a key factor in the engagement with the intervention. As part of ORBIT, ongoing implementation work is investigating how this system may be best positioned if it were to be part of routine care. It should be noted that all the research costs, such as a suite of outcome measures, sophisticated analytical databases, randomisation systems are unlikely to be required if the intervention was delivered in routine care. Thus, the short-term research costs should be balanced against a longer-term societal benefit. Blending digitally delivered interventions has previously been reported as particularly advantageous for delivering treatment, offering the opportunity to improve access to cost-effective treatments that are efficacious in supporting behavioural change [44]. Here we demonstrate that blending technology-supported procedures (i.e. referrals, outcome measure completion and the intervention) with research staff to deliver trials is likely to be a promising avenue for trial methodology.

## Conclusion

Recruitment, retention/engagement and trial staff are key factors for successful trial delivery and are likely to be the biggest risk factors in trial completion. Utilising an example of an online-delivered trial with human support (ORBIT) we demonstrate how a blended human/online approach may be particularly advantageous in facilitating trial delivery, particularly in over-stretched and under-resourced services or in hard to reach populations who are comfortable in using technology (such as youth populations). Potential benefits include flexibility in the timing and location of delivery of interventions and measures, partially or fully automated data collection and ability to recruit over a large geographical area whilst maintaining a rapport delivered by human support. We also advocate that trials are adequately costed in the initial bid development phases to provide the necessary infrastructure and staff to support delivery. Further research is required to improve trial delivery and reduce waste of human and economic resources.

## Data Availability

The datasets generated and/or analysed during the current study are held by the NIHR HTA. Any requests for anonoymised data should be made to the NIHR HTA.

## Abbreviations

AHRQ: Agency for Healthcare Research and Quality
CTIMP: Clinical Trial of an Investigation Medicinal Produc
GPs: General practitioners
HTA: Health Technology Assessment
NHS: National Health Service
NICE: National Institute of Health and Care Excellence
NIHR: National Institute of Health Research
ORBIT: Online Remote Behavioural Intervention for Tics
PIC: Patient Identification Centre
RCTs: Randomised controlled trials
STEPS: Strategies for trial enrolment and participation study
